# Transcatheter Bioprosthetic Aortic Valve Dysfunction: What We Know So Far

**DOI:** 10.3389/fcvm.2019.00145

**Published:** 2019-10-04

**Authors:** Fadi Sawaya, Troels H. Jørgensen, Lars Søndergaard, Ole De Backer

**Affiliations:** ^1^The Heart Center, Rigshospitalet, Copenhagen University Hospital, Copenhagen, Denmark; ^2^Department of Cardiology, American University of Beirut Medical Center, Beirut, Lebanon

**Keywords:** aortic valve replacement, transcatheter, valve dysfunction, valve deterioration, thrombosis, endocarditis

## Abstract

Transcatheter aortic valve replacement (TAVR) is an established alternative to surgical valve replacement for patients with severe aortic stenosis (AS) and increased surgical risk. On the basis of the favorable outcomes of recent randomized clinical trials conducted in intermediate and low risk populations, TAVR is expected in the near future to be offered to patients not only at lower surgical risk, but also with longer life expectancy. In this particular subset, the long-term durability of the bioprosthetic valve is of critical importance. The European Association of Percutaneous Cardiovascular Interventions (EAPCI), the European Society of Cardiology (ESC), and the European Association for Cardio-Thoracic Surgery (EACTS) recently introduced standardized criteria to define structural valve deterioration (SVD) and valve failure of transcatheter and surgical aortic bioprosthesis—this with the aim to generate uniformity in data reporting in future studies assessing long-term durability of aortic bioprosthesis. On this background, the aim of this article is to review the definition, incidence and predictors of transcatheter bioprosthetic valve dysfunction, including structural and non-structural valve deterioration (SVD/NSVD), valve thrombosis, and endocarditis.

## Introduction

The clinical impact of transcatheter aortic valve replacement (TAVR) has been important by addressing the need for a therapeutic treatment in selected inoperable patients with symptomatic severe aortic stenosis (AS), but also as an alternative to surgical aortic valve replacement (SAVR) in patients at increased surgical risk. Based on large randomized controlled trials (RCTs) comparing TAVR to SAVR in patients with severe AS and intermediate or lower surgical risk (1–3), both the guidelines from the European Society of Cardiology (ESC) and the European Association for Cardio-Thoracic Surgery (EACTS) as well as the guidelines from the American Heart Association (AHA) and American College of Cardiology (ACC) were updated in 2017, upgrading the indication for TAVR to patients with symptomatic severe AS and intermediate surgical risk (4–6). Within the past decade, national registries have reported a steady growth in the annual number of TAVR procedures performed (7–10) and a decrease in the mean age of patients undergoing TAVR (8). The anticipation of treating AS patients with a longer life expectancy by means of TAVR has intensified the concerns about the durability of transcatheter heart valves (THVs).

Recently, two randomized clinical trials including patients with low surgical risk were presented. The Evolut Low Risk trial reported that TAVR with a self-expanding THV was non-inferior to SAVR with regard to all-cause mortality or disabling stroke within 2 years of the procedure (11). The PARTNER 3 trial reported that TAVR with a balloon-expandable THV was superior to SAVR with regard to the 1-year risk of all-cause mortality, stroke, or re-hospitalization (12). The mean age in both trials was 74 years of age. Considering these results, it is likely that the indication for TAVR will not only expand to patients with lower surgical risk but also to patients at younger age than currently treated by TAVR. Consequently, with the increased life expectancy of these patients, it is more likely that they will outlive their implanted THV. There are only few data regarding long-term THV durability. Assessment of valve function in the early randomized TAVR trials and registries have consistently shown preserved valve function up to 5 years after TAVR (13, 14). However, it is well-documented that valve degeneration with surgical aortic bioprostheses is usually not seen until 5–10 years post-procedure (15).

**Figure 1:**
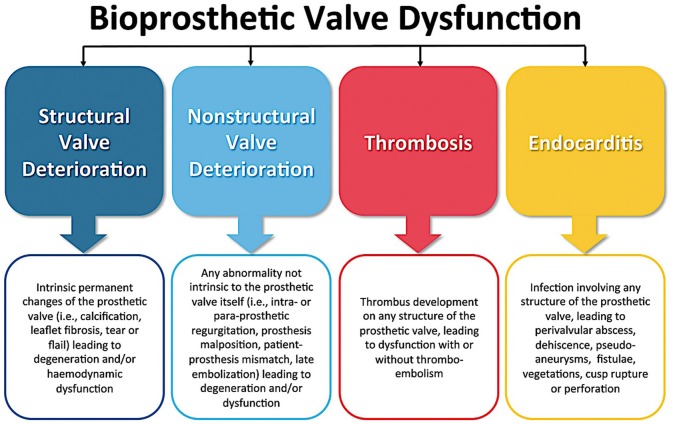
Causes of bioprosthetic valve dysfunction. Figure used with permission from Oxford University Press (16).

The aim of this article is to review the definition, incidence and predictors of transcatheter bioprosthetic aortic valve dysfunction, including structural and non-structural valve deterioration (SVD/NSVD), valve thrombosis, and endocarditis (Figure 1).

## Structural Valve Deterioration

The durability of surgical aortic bioprosthesis has been more extensively described compared with THVs, mainly due to SAVR being performed for a longer time. However, previous studies reporting on surgical heart valves have tended to use the need for re-intervention as a clinical endpoint for SVD (17–20)—resulting in a risk of under-estimating the incidence of SVD as some patients might have become too frail over time to undergo redo-SAVR, even though the surgical aortic bioprosthesis might have been deteriorated (20, 21). Some echocardiographic criteria for defining SVD have been applied in individual studies (22); however, the lack of a consensus definition for SVD limits the possibility for comparison across studies.

Efforts have been made to standardize the definition of SVD for both transcatheter and surgical bioprostheses. Building on existing definitions for SVD, a consensus statement from the European Association of Percutaneous Cardiovascular Interventions (EAPCI)—endorsed by the ESC and the EACTS—was published in 2017 (16). SVD was classified into either morphological or moderate and severe hemodynamic SVD based on the severity of THV stenosis and/or regurgitation (16). The VIVID (Valve in Valve International Data) group published a similar but more staged classification for SVD, Table 1 (22).

**Table 1:** Proposed definitions of structural valve deterioration (SVD).

**Valve Academic Research Consortium (VARC)-2 (2013)**
**Structural Valve Deterioration (SVD)** Valve related dysfunction (any of the following): ∘ Mean gradient ≥ 20 mmHg ∘ Effective orifice area (EOA) ≤ 0.9–1.1 cm^2^ ∘ Doppler Velocity Index (DVI) < 0.35 m/s ∘ Moderate or severe prosthetic valve regurgitation Requiring repeat procedure aortic valve replacement
**European Association of Percutaneous Cardiovascular Interventions (EAPCI) endorsed by ESC and EACTS (2017)**
**Morphological SVD** (any of the following): Leaflet integrity, structure, or function abnormality (i.e., flail, pathological thickening, calcification, impaired mobility causing central regurgitation, or valvular stenosis) Strut/frame abnormality (i.e., fracture)
**Moderate hemodynamic SVD** (any of the following): Mean gradient ≥ 20 and <40 mmHg Mean gradient ≥ 10 and <20 mmHg change from baseline Moderate intra-prosthetic aortic regurgitation, new or worsening (>1/4) from baseline
**Severe hemodynamic SVD** (any of the following): Mean gradient ≥ 40 mmHg Mean gradient ≥ 20 mmHg change from baseline Severe intra-prosthetic aortic regurgitation, new or worsening (>2/4) from baseline
**Valve-in-valve international data registry (VIVID) investigators (2018)**
**SVD stage 0** No significant change from immediate post-implantation
**SVD stage 1** Morphological leaflet abnormality without significant hemodynamic changes
**SVD stage 2S** Moderate stenosis (mean gradient ≥ 20 and <40 mmHg) ≥10 mmHg increase from baseline status concomitant with increase in EOA and DVI Thrombotic leaflet thickening excluded
**SVD stage 2R** Moderate regurgitation Exclude that paravalvular regurgitation is main component
**SVD stage 2RS** Stage 2S and 2R
**SVD stage 3D** (any of the following) Severe stenosis (mean gradient ≥ 40 mmHg) Severe regurgitation

SVD is commonly defined as an intrinsic permanent change of the bioprosthesis due to leaflet calcification, thickening, pannus formation, tear, or disruption. The resulting deterioration leads to stenosis and/or intra-prosthetic regurgitation (16, 21–23).

A study including 2,659 patients undergoing SAVR reported that the time to deterioration of surgical bioprostheses was similar regardless of stenotic and/or regurgitation etiology (21). Further, younger age was found as a significant risk factor for early SVD (17, 21). The increased risk of SVD in younger patients is thought to be mediated by a higher metabolic rate and a stronger immunological response to the implanted bioprosthetic heart valve (19). Other factors that have been found to increase the risk of SVD of aortic bioprostheses are patient-prosthesis mismatch (PPM), dialysis, hyperparathyroidism, and diabetes (17, 19, 24, 25).

In addition, it has been hypothesized that several risk factors of SVD might specifically apply to THVs. The need to crimp the THV into a delivery catheter may theoretically damage the bioprosthetic leaflet tissue, thereby impairing THV durability (26). During TAVR, it is not aimed to align the commissures of the THV to those of the native aortic valve, and commissural misalignment might increase the stress on the leaflets and has been reported to increase the risk of mild intra-prosthetic regurgitation (27). Lastly, there is no data on the impact of elliptical THV geometry due to calcified native aortic annulus or incomplete expansion of the THV due to oversizing.

So far, there is only limited data available on long-term durability of THVs, as those patients treated by TAVR in the early years of the procedure were typically elderly and frail patients. Consequently, the expected and observed medium-term survival rate of these initially treated TAVR-patients is relatively low, Table 2. As abovementioned, age also likely plays a crucial inverse role in the rate of SVD. This further limits the possibility to predict the rate of SVD based on the currently treated patients whom are likely older than the future TAVR patients.

**Table 2:** Incidence of structural valve deterioration of transcatheter heart valves.

**References (definition used)**	**Valve type—number**	**5-year survival**	**Median F/U (months)**	**Structural valve deterioration**
Aldalati et al. (28) (EAPCI/ESC/EACTS)	Sapien: 52 Sapien XT: 156 Sapien 3: 51 Lotus: 6 Jena valve: 2 Others: 2	45%	33	Moderate/severe: 33% (6.5 year estimate)
Gerckens et al. (29) (VARC-2)	CoreValve: 996	49%	36	Moderate/severe: 2.6%
Eltchaninoff et al. (30) (EAPCI/ESC/EACTS)	Cribier: 79 Sapien: 83 Sapien XT: 216	32%	36	Moderate/severe: 3.2% (8 year estimate)
Barbanti et al. (31) (N/A)	CoreValve: 353	45%	47	Moderate/severe: 3.7%
Gleason et al. (32) (EAPCI/ESC/EACTS)	CoreValve: 391	N/A	50	Severe: 0.8%
Toggweiler et al. (33) (VARC-1)	Cribier: 49 Sapien: 39	35%	60	Moderate: 3.4%
Mack et al. (34) (N/A)	Sapien: 348	32%	60	Moderate/severe central regurgitation: 0.7%
Blackman et al. (35) (EAPCI/ESC/EACTS)	Sapien: 45 Sapien XT: 35 CoreValve: 149 Portico: 4	N/A	69	Moderate: 8.7% Severe: 0.4%
Panico et al. (36) (EAPCI/ESC/EACTS)	CoreValve: 278	45%	70	Moderate/severe: 3.6%
Søndergaard et al. (37) (EAPCI/ESC/EACTS)	CoreValve: 139	72%	72	Moderate: 3.6% Severe: 0.7%
Holy et al. (38) (EAPCI/ESC/EACTS)	CoreValve: 152	50%	75	Severe: 0%
Didier et al. (39) (EAPCI/ESC/EACTS)	Self-exp.: 1,413 Balloon-exp.: 2,774	39%	N/A	Moderate: 10.8% Severe: 2.5% (5 years estimate)
Barbanti et al. (40) (EAPCI/ESC/EACTS)	CoreValve: 238 Sapien XT: 48	55%	N/A	Moderate: 5.9% Severe: 2.4% (8 years estimate)

Only few data exist on controlled comparison of SVD between surgical and transcatheter aortic bioprosthesis. In general, these studies find the medium-long term risk of severe SVD to be low, ranging between 0 and 2.5% in studies with 4–8 years of follow-up (32, 35, 37–40). In the PARTNER-1 trial, there was no SVD reported at 5 years—although only 15 patients remained alive (13). In the CoreValve High Risk Pivotal trial, severe SVD was noted in 1.7% and 0.8% of patients at 5 years after SAVR and TAVR (*p* = 0.32), respectively (32). The standardized criteria of SVD from EAPCI/ESC/EACTS were recently also applied to the NOTION trial—a RCT randomizing AS patients at lower surgical risk to SAVR or TAVR. This patient population had a lower mortality rate as compared to contemporary RCTs in high-to-intermediate risk patients. The risk of SVD through 6 years for surgical and transcatheter bioprostheses was 24.0 and 4.8% (*p* < 0.0001), respectively (37). This difference was mainly driven by moderate SVD in the SAVR group and severe SVD was observed in 3.0% vs. 0.7% in the SAVR vs. TAVR group, respectively (*p* = 0.21) (37).

## Non-structural Valve Deterioration

Non-SVD is a bioprosthetic abnormality due to extrinsic factors such as PPM, paravalvular regurgitation (PVR), device malpositioning, or abnormal frame expansion. The presence of non-SVD is not an intrinsic deterioration of the bioprosthesis or leaflets—however, it might mediate early development of SVD (16, 22, 41).

Following SAVR, the prevalence of moderate PPM ranges from 20 to 70% and that of severe PPM from 2 to 20% (42, 43). TAVI is associated with a lower prevalence of (especially severe) PPM as compared to SAVR (44, 45). Among THVs, self-expanding valves with supra-annular design are generally associated with a lower prevalence of PPM as compared with balloon-expandable valves. Patients with PPM have worse symptoms and exercise capacity, higher rate of heart failure re-hospitalization, and increased mortality after SAVR as compared with patients with no PPM (42, 46). PPM is also associated with quicker structural degeneration of surgical aortic bioprostheses (24, 47). Consequently, TAVR could be the preferred choice of treatment for patients with a small annulus (valve size < 23 mm) in whom PPM can be anticipated in case of SAVR.

On the contrary, THVs more frequently have PVR. However, with the newer generation THVs—with often an additional sealing skirt around the valve prosthesis stent frame—these PVR rates have come down to a range of 1–3% for moderate PVR and 29–36% for mild PVR in the latest low-risk TAVR trials (11, 12). The impact of mild PVR on left ventricular function, symptoms, and long-term mortality in lower risk patients with longer life expectancy is still unknown—however, data from the PARTNER-1 trial have suggested decrease survival in this subset (13).

Finally, whether abnormal stent frame expansion—as sometimes observed in case of THV in heavily calcified or bicuspid valves—has a negative impact on valve durability is unknown. In an *ex-vivo* bench study by Sathananthan et al., it was shown that excessive THV overexpansion may be associated with impaired hydrodynamic function, acute leaflet failure, and reduced durability. Smaller valves may be at greater risk with overexpansion than larger valves. Similar, THV undersizing can cause leaflet pin wheeling and reduced durability (48).

In conclusion, future TAVR studies including patients with longer life expectancy and bicuspid valve anatomy are needed to answer these remaining open questions.

## Valve Thrombosis

Clinical valve thrombosis after TAVR typically presents with an increase of transvalvular gradient and symptoms of heart failure caused by obstructing thrombus in the THV. On the other hand, subclinical leaflet thrombosis is an incidental finding on 4DCT or TEE imaging, which does not cause symptoms or elevated transvalvular pressure gradients outside the normal range.

In a study by Bourguignon et al. (21) with long-term follow-up data on 2,659 Carpentier-Edwards Perimount valves in the aortic position, no single case of clinical valve thrombosis was reported with a median follow-up of 6.7 years (21). In accordance, there was no clinical valve thrombosis observed in the NOTION lower-risk trial—neither in the TAVR nor in the SAVR population—with a follow-up of up to 6 years (37). In two retrospective analyses, the prevalence of clinical valve thrombosis was reported to be 0.6 and 2.8% after TAVR (49, 50), whereas the prevalence of subclinical leaflet thrombosis has been reported to be as high as 15–35% in studies assessing this phenomenon by means of TEE and/or 4DCT cardiac imaging (51–53).

Although usually an incidental finding, there has been a concern that subclinical leaflet thrombosis may progress into clinical valve thrombosis, cause stroke or other thromboembolic events, and/or impair the durability of the THV.

Reports on a potential association between subclinical leaflet thrombosis and stroke/transient ischemic attack (TIA) have raised concerns. In the SAVORY and RESOLVE registries, subclinical leaflet thrombosis with reduced leaflet motion was associated with increased incidence of TIA (53). In contrast, a prospective trial—including 4DCT or echocardiography in 434 patients that underwent TAVR—did not show any increased stroke risk at 3 years of follow-up in those patients diagnosed with (possible) subclinical leaflet thrombosis (54). One meta-analysis, although involving a limited number of retrospective studies, also reported an overall odds ratio of 3.38 (95% CI: 1.78–6.41, *P* < 0.001) for cerebrovascular events in case of hypo-attenuation affecting motion (HAM) as compared with hypo-attenuation affecting leaflet thickening (HALT) only—thereby suggesting an impact of “thrombus burden” on the risk for neurological events (55). However, it should be kept in mind that all these reports are based on retrospective data and often there is a very long temporal separation between the neurological event and the cardiac imaging showing subclinical leaflet thrombosis.

**Figure 2:**
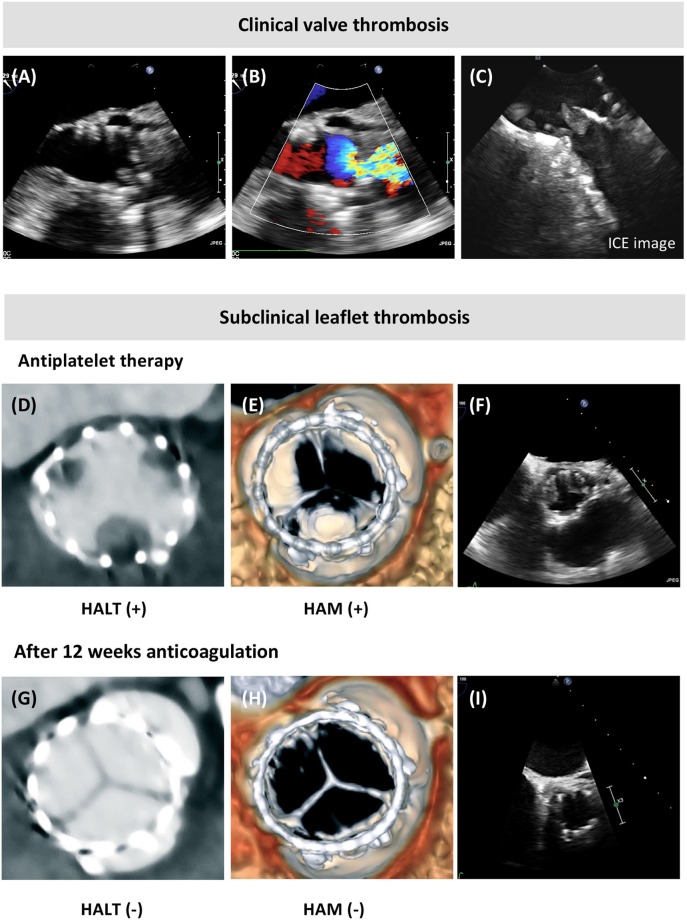
Clinical valve thrombosis and subclinical leaflet thrombosis. **(A,B)** Transesophageal echocardiography (TEE) showing valve thrombosis and turbulent color flow over the transcatheter aortic bioprosthesis in a patient presenting with an elevated mean transvalvular gradient at transthoracic echocardiography (TTE, 37 mmHg) and dyspnea NYHA class 3–4, and this few years after TAVR. **(C)** The thrombotic mass at the aortic side of the prosthetic leaflets was confirmed by intracardiac echocardiography (ICE). **(D,E)** Incidental finding of hypoattenuating leaflet thickening (HALT) at the base of the transcatheter heart valve leaflets, with hypoattenuation affecting motion (HAM) visible in systole in the volume-rendered 4D computed tomography (4DCT) images; **(F)** this reduced leaflet motion of two leaflets was confirmed by TEE. **(G–I)** Resolution of the leaflet thickening and reduced leaflet motion following 3 months of anticoagulation treatment, as shown by 4DCT and TEE imaging.

Anticoagulation seems to be preventive for development of both clinical valve thrombosis and subclinical leaflet thrombosis, whereas single or dual APT does not have this protective effect. In accordance, treatment with anticoagulation seems to have—at least temporarily—beneficial effects on restoration of leaflet motion and transvalvular gradients in case of THV thrombosis (50, 51, 56) (Figure 2). In addition, Del Trigo et al. reported that absence of anticoagulant therapy at hospital discharge was an independent predictor of hemodynamic valve deterioration after TAVR (57).

Finally, although subclinical leaflet thrombosis seems to be more common after TAVR as compared with SAVR, medium-term durability of THVs has been reported to be non-inferior to surgical aortic bioprosthesis in several large randomized trials (32, 34). Whether, subclinical leaflet thrombosis may be a precursor and/or predictor of valve dysfunction on the longer-term is unknown. Long-term follow-up data on THV and surgical bioprosthesis—including leaflet-imaging studies—will be needed in order to give an answer to this question.

## Endocarditis

Another important reversible mechanism of non-SVD is the development of valve failure due to infective endocarditis. In the NOTION lower-risk trial, the risk of infective endocarditis was similar after SAVR (5.9%) as compared to TAVR (5.8%) after 6 years of follow-up (37). In accordance, Butt et al. (58) recently reported long-term follow-up data from a Danish nationwide observational study comprising 2,632 TAVR patients and 3,777 matched SAVR patients. During a mean follow-up of 3.6 years, 115 patients (4.4%) with TAVR and 186 patients (4.9%) with SAVR were admitted with infective endocarditis. The median time from procedure to infective endocarditis hospitalization was 352 days in the TAVR group and 625 days in the SAVR group. The cumulative 5-year risk of infective endocarditis was 5.8% and 5.1% in the TAVR and SAVR population, respectively—hence, the long-term risk of infective endocarditis was similar following TAVR and SAVR (58).

In a large collaborative study, a total of 250 cases of infective endocarditis occurred in 20,006 patients after TAVR (incidence, 1.1% per person-year; 95% CI, 1.1–1.4%; median age, 80 years; 64% men). Median time from TAVR to infective endocarditis was 5.3 months [interquartile range (IQR), 1.5–13.4 months]. The characteristics associated with higher risk of progressing to infective endocarditis after TAVR was younger age [78.9 years vs. 81.8 years; hazard ratio (HR), 0.97 per year], male sex (62.0% vs. 49.7%; HR, 1.69), diabetes mellitus (41.7% vs. 30.0%; HR, 1.52), and moderate to severe aortic regurgitation (22.4% vs. 14.7%; HR, 2.05). Patients who developed endocarditis had high rates of in-hospital mortality (36%) and 2-year mortality (66.7%) (59).

The same prophylactic measurements to prevent infective endocarditis have to be implemented following AVR with a surgical or transcatheter aortic bioprosthesis. In case of suspicion of prosthetic valve endocarditis in a TAVR patient, the diagnosis with TEE may sometimes be challenging—in such cases, use of intracardiac echocardiography (ICE) and PET-CT could be considered (58, 60). In case of prosthetic valve endocarditis following TAVR, the therapeutic options are most of the time limited to antibiotic medical treatment. Limited data from the Copenhagen group indicate that such conservative approach is associated with a 22% mortality rate. As reported by Olsen et al. (60), 17 out of 18 TAVR patients with infective endocarditis were treated conservatively and one with surgery. Four patients (22%) died from endocarditis or complications to treatment, two of those (11%) during initial hospitalization for prosthetic valve endocarditis (60).

## Conclusions

With the expansion of TAVR to patients with longer life expectancy, data on long-term THV durability are essential. The first studies reporting on SVD up to 8 years after TAVR show low rates of THV degeneration. Importantly, the release of standardized definitions on bioprosthetic valve dysfunction represents a fundamental step in allowing obtaining a better insight into its real incidence.

## Author Contributions

All authors listed have made a substantial, direct and intellectual contribution to the work, and approved it for publication.

### Conflict of Interest

The authors declare that the research was conducted in the absence of any commercial or financial relationships that could be construed as a potential conflict of interest.
